# Supplementary material for: An agent-based model of the spread of behavioural risk-factors for cardiovascular disease in city-scale populations
Source: PLoS One. 2024 May 28;19(5):e0303051. doi: 10.1371/journal.pone.0303051 (PMC11132484; doi:10.1371/journal.pone.0303051)
Supplement: Attachment — Submitted filename: Response to reviewers.docx [file pone.0303051.s001.docx]

**Response to Reviewers**

Paper Title: An Agent-Based Model of the Spread of Behavioural Risk-Factors for Cardiovascular Disease in City-Scale Populations
Authors: James Archbold, Sophie Clohessy, Deshani Herath, Nathan Griffiths, and Oyinlola Oyebode

We thank the Reviewers for their comments, and the Editor for their consideration. In the table below we outline our responses to the individual comments and the main changes to the manuscript. We have also made minor changes to correct typographical errors and improve clarity.

| **Reviewer 1** |  |
| --- | --- |
| Thank you for the opportunity to review the article “An Agent-Based Model of the Spread of Behavioural Risk-Factors for Cardiovascular Disease in City-Scale Populations. It is a well-written paper strongly founded on robust predictive modelling. I greatly appreciate the efforts of the authors. However, I have a few suggestions to strengthen the article further. I have a few suggestions and observations related to the model | Thank you for your helpful suggestions. |
| 1. I see a possibility of extending the simulation with more factors, like a. Common mental disorders like stress, anxiety, and depression have shown a significant association with behavioural risk factors. Therefore, how does the model address these intervening factors? It is not clear from the manuscript. | We didn’t include these factors because we focused on the four key behaviours that impact on CVD, and used an algorithm for generating CVD risk based on those four behaviours and intermediate physiological risk factors (explained in the subsection “CVD Risk”). We did not have the data to allow us to model the effect of common mental disorders, and this was not the focus of the paper.  We added a sentence in the “CVD Risk” section, as follows:  “We use only the core parameters of QRISK3, and all optional modifications are ignored since our ABM does not include the corresponding factors. These could be introduced in a future extension of the model, if relevant data were identified.”  We agree that it may be possible to extend the model in the future by considering that people with mental health conditions are more likely to engage in risky behaviours, and more likely to develop CVD. There is also some evidence that mood spreads through social networks (although this may not extend to mental illness). This could be done if we were able to identify relevant data. We have now added this to the list of possible extensions in the discussion (along with a list of other clinical inputs in QRISK3 that we could incorporate if data was available). Specifically, we have added, “Agents could express other risk-factors including behaviours, or clinical risk-factors for CVD such as mental illness, lupus or rheumatoid arthritis.” |
| b. The genetic loading of CVD is not included in the model, as it could be a confounder. To my knowledge, behavioral aspects are only one of the various genetic, cognitive and social risk factors of CVD | Data doesn’t exist to allow us to assign genetic vulnerability to each agent, and this would be computationally complex. We are not aiming to develop a fine-grained simulation that predicts individual CVD, but each agent has a baseline risk of CVD (regardless of the behavioural risks assigned), which accounts for genetic risk and other factors which are not explicitly modelled. |
| 2. Comparing with a network model like the Stochastic Block Model (SBM)/ or a centrality measure that considers influence strength would enhance the model's robustness by capturing the higher likelihood of interactions within the same neighbourhood. | We have added discussion, in the “Spreading Models” and “Networks” sections, of how our network modelling approach relates to the SBM, and have clarified why centrality measures are not appropriate. We use Newman-Watts-Strogatz small-world networks [42] since such networks have been shown to have similar properties to real-world networks [37-41]. There is no need for the ABM to consider centrality or other measures of influence directly (although we have added brief discussion of them in the paper), since we are modelling a population of agents as a whole rather than selecting a “seed set” of individuals. Thus, in using a small-world network, the more influential individuals (i.e., those of high degree or centrality) will naturally exert more influence in the simulation. |
| 3. The manuscript does not account for temporal dynamics, such as the short-term and long-term effects of behaviours on cardiovascular health. | We have made this explicit in the “CVD Risk” section by adding the following text: “We are not able to account for short and long-term effects of a behaviour, so, for example an agent that is an ex-smoker experiences the same increase in their risk of CVD whether it was a current smoker in the previous time step, or several time steps previously. Similarly, an agent consuming 50+ units of alcohol a week experiences the same increase in their risk of CVD whether they have expressed this behaviour for one or ten time steps. There is data available that could inform a future extension of the model to take this into account.” |
| 4. Conduct sensitivity analyses to assess the impact of varying parameters on model outcomes | The “Varying Influence: Modelling Sensitivity” section has been updated to better highlight the impact of varying parameters. Note that we focus on varying the behaviour factors, not the population factors, in order to maintain relevance to a general UK population. |
| **Reviewer 2** |  |
| This is a clear and well written text that describes an agent based model of the reinforcement or mitigation of CVD behavioral risk factors by spousal, household, friendship and workplace networks. | Thank you for your helpful suggestions |
| The model is well described and compelling with one drawback that is leading me to recommend major revision. The various networks are created during initialization and then remain static over the course of the simulated 10 year run. The static nature of the networks seems perhaps reasonable for spouse and household, but less so for friendship and workplace networks. The authors address this shortcoming in the "Limitations of the ABM Model" section, but not satisfactorily. The strength and central focus of the paper is the network mediated influence and stating that "the population in the ABM can be considered as a cohort that persists throughout the simulation process" undermines that focus. If the CVD behavioral risk factors are network mediated then the static nature cannot be dismissed so easliy. More needs to be said about why the static nature of the networks doesn't break the model, perhaps with additional experiments that do change the networks to illustrate what sort of effect, in the abstract, this might have. In addition, workplace assignment as described in the synthetic population literature may also offer some insight into workplace turnover. With respect to dynamic networks, the authors may also want to look at "ERGMs" ([https://eehh-stanford.github.io/SNA-workshop/ergm-intro.html](https://eur01.safelinks.protection.outlook.com/?url=https%3A%2F%2Feehh-stanford.github.io%2FSNA-workshop%2Fergm-intro.html&data=05%7C02%7Co.oyebode%40qmul.ac.uk%7C1522617f0de44c45010008dc217fd293%7C569df091b01340e386eebd9cb9e25814%7C0%7C0%7C638422083627112800%7CUnknown%7CTWFpbGZsb3d8eyJWIjoiMC4wLjAwMDAiLCJQIjoiV2luMzIiLCJBTiI6Ik1haWwiLCJXVCI6Mn0%3D%7C0%7C%7C%7C&sdata=vmsZl%2FvzL%2BKZNkxmVs3uv8H8quSWWhpDgYgq5hpLce4%3D&reserved=0)). More specific comments follow. | We do not model the dynamic nature of friendships or workplace contact groups since there is a lack of real-world data on how these evolve. Given the lack of data, the main options would be to either to rewire randomly or to grow the network using a method such as preferential attachment (as per the Barabási-Albert random graphs [40]). While rewiring has been used in the literature to model dynamic networks, this typically causes networks to lose their small-world or scale-free nature. Using preferential attachment would involve inserting new agents into the simulation and, as discussed in the “Limitations of the ABM Model” section we do not have real-world data on how to do this.  We have extended the discussion of the issues with creating the friendship network and its potential dynamic nature in the “Friendship” section, discussing generation of the network, and the “Limitations of the ABM Model” section. We have also discussed how our approach relates to ERGMs (and the SBM) in the “Friendship” section. |
| In the Related Work section, some brief discussion of synthetic population construction would be useful. | Thank you – this change has been made. |
| line 38. "the evolution", maybe better as "their evolution" or "the evoluion of ...". | Thank you – this change has been made. |
| l. 65 The two influence models need citations. They are cited later, but this is their first mention, I think. | Thank you – this change has been made. |
| In the "Basic Agent Attributes" section, it would be useful to explicitly mention that the agent's have the risk factor attributes as well as age, sex, and socio-economic status. This is obvious once the model is further described, but would be good here as well to avoid any confusion. With respect to the attributes, the authors state "these attributes have a significant effect on an agent’s likelihood of developing CVD, and are considered in combination with the impact of the behavioural risk factors described below". Further in the text, when creating the population and assigning CVD risk factors, age and sex are used, but it's unclear how socio-economic status (IMD) effects CVD in the model. | We have updated the “Basic Agent Attributes” section to improve the clarity of our agent description, as well as to make explicit the contribution of socio-economic status / IMD to the model. |
| With respect to behavior spread, why 1 year time steps? | We used 1 year time steps as a natural unit, since we model agents’ age and several of our data sources use 1 year granularity (e.g., QRISK3 uses age in years as a factor, and the ONS data is indexed by year). |
| l. 371 - no need to unpack CSV, already seen on page 12. | Thank you for spotting this, it is now corrected. |
| l. 408 - Missing dash in Hippisley Cox. This occurs in other places as well. | Thank you for spotting this, it is now corrected. |
| Calibration Section - some text about the stochastic nature of the model, and the range of variation between runs with different seeds, and how many runs are necessary to characterize the model's output would be useful, assuming this applies. | In response to this point, we have included the standard deviation in more of our experiment results tables and added a new paragraph in the “Experimental Parameters” section to discuss our approach to dealing with the inherently stochastic nature of the model. |
| Results - some text about why some age ranges do better / worse that others would help to explain the results. | If this comment is asking about why some age ranges have a higher rate of CVD than others, the answer is that age is the biggest risk-factor for CVD. We have added the following sentence in the “Baseline Model” section to reflect this. “This shows the expected epidemiological patterns for CVD risk, i.e.: higher rates for older age-groups and for men compared with women, which are well-known trends.”  If this comment is asking about why some age ranges do better in terms of the rates more closely matching observed data (e.g., for 55-65 year olds) this may be to do with risk-factors specific to this particular age-range that we are not modelling well. We have added the following text: “The modelled data is slightly further from the observed data for the middle age-groups (ages 55-64), although still acceptable, which may suggest there are specific risk-factors in these age-groups that we do not currently model, or model quite as well.” |
| Intervention - why does the baseline model against which intervention is applied have no workplace influence. More accurate / realistic would be a calibrated real-world reasonable level of workplace influence that is then modified by the intervention, unless the experiment is only to show that an intervention can have an effect in the abstract. If it's the latter, then that should be made more explicit. | We added the workplace network as a feature in the ABM, but did not have the data to calibrate workplace influence in the baseline case. Instead, the aim of including the workplace network was to illustrate the potential nature of interventions, and show how the workplace network might impact behaviour. If suitable real-world data becomes available, the baseline model could be calibrated to include an appropriate level of workplace influence. By including the ability to model workplace influence we aim to support investigation into how this factor might impact the spread of behaviour.  We have added clarification in the “Workplace Contacts” and “Simulation Parameters” sections. |
| l. 507 -- isn't the maximum absolute difference 7.86? | Thank you for highlighting this – the text has been updated. |
| Is citation 39, the same as 3? | Thank you for highlighting this – we have unified these references. |
